# Photodynamic therapy of C3H mouse mammary carcinoma with haematoporphyrin di-ethers as sensitizers.

**DOI:** 10.1038/bjc.1987.98

**Published:** 1987-05

**Authors:** J. F. Evensen, S. Sommer, C. Rimington, J. Moan

## Abstract

Haematoporphyrin di-ethers were synthesized and tested as sensitizers for photodynamic therapy of C3H/Tif mammary tumours in mice. Growth curves of the tumours were determined by measuring the tumour volume. The animals were given 25 mg porphyrins kg-1 body weight i.p. and 24 h later exposed to 135 J cm-2 of 630 nm light at a fluence rate of 150 mW cm-2. The sensitizing efficiency of the ethers was measured in terms of the increase in growth time of the treated tumours, as compared with that of untreated controls, needed to reach a volume 5 times larger than that at the time of the treatment. This sensitizing efficiency increased with decreasing polarity, i.e. in the order di-methyl ether, di-propyl ether, dibutyl ether and di-amyl ether. Haematoporphyrin di-amyl ether was more efficient than haematoporphyrin derivative and insignificantly less efficient than photofrin II (DHE). This was true for sensitization of both tumours and normal tissue.


*Br. J. Cancer* (1987), **55**, 483–486 © The Macmillan Press Ltd., 1987

### Photodynamic therapy of C3H mouse mammary carcinoma with haematoporphyrin di-ethers as sensitizers

J.F. Evensen, S. Sommer, C. Rimington & J. Moan

*Institute for Cancer Research, The Norwegian Radium Hospital, 0310 Oslo 3, Norway.*

**Summary** Haematoporphyrin di-ethers were synthesized and tested as sensitizers for photodynamic therapy of C3H/Tif mammary tumours in mice. Growth curves of the tumours were determined by measuring the tumour volume. The animals were given 25 mg porphyrins $kg^{-1}$ body weight i.p. and 24 h later exposed to 135 $J cm^{-2}$ of 630 nm light at a fluence rate of 150 $mW cm^{-2}$. The sensitizing efficiency of the ethers was measured in terms of the increase in growth time of the treated tumours, as compared with that of untreated controls, needed to reach a volume 5 times larger than that at the time of the treatment. This sensitizing efficiency increased with decreasing polarity, i.e. in the order di-methyl ether, di-ethyl ether, di-propyl ether, di-butyl ether and di-amyl ether. Haematoporphyrin di-amyl ether was more efficient than haematoporphyrin derivative and insignificantly less efficient than photofrin II (DHE). This was true for sensitization of both tumours and normal tissue.

Haematoporphyrin derivative, HpD, and its purified version Photofrin II, also called DHE, are porphyrin preparations that are being tested clinically for use as sensitizers in photodynamic cancer treatment (PDT). So far several promising reports on the efficacy of this treatment have been published (Dougherty, 1985 and references cited therein). The complete chemical composition of the active fraction of HpD and DHE has not been finally resolved, although it has been proposed that it consists of dihematoporphyrin ethers or esters (Bonnett & Berenbaum, 1983; Dougherty, 1984; Kessel *et al.*, 1985). Neither is it known why certain components of these porphyrin preparations are selectively retained by tumours compared with many normal tissues. It has been assumed that polarity and aggregation properties may play important roles (Moan, 1986). This led us to prepare and test several porphyrin di-ethers with chromatographic properties, and hence polarities, in the range of those of the tumour-localizing fraction of HpD and DHE. These compounds have a known chemical structure and with one exception we found that they could be purified to the degree desired. It is a major drawback for the clinical and experimental use of HpD and DHE that they contain so many chromatographically different components (Bonnett *et al.*, 1980; Moan & Sommer, 1984). Even though many of these components may be isomeric structures of the same porphyrin, one would prefer to use a single, well defined compound of known structure.

### Materials and methods

Photofrin II (DHE, lot PC 218) and Photofrin I was obtained from Photomedica, Inc., Raritan, N.J. Hematoporphyrin (Hp) di-hydrochloride from Koch Light was used in the preparation of HpD, according to the method of Lipson *et al.* (1961). This product was similar, both with respect to chromatographic properties and sensitizing efficiency, to Photofrin I from Photomedica. Since the sensitizing efficiency of Hp was to be tested, we ensured by chromatographic analysis (RP 18 column eluted with a water/methanol gradient and both absorbance and fluorescence detection as described by Sommer *et al.*, 1984) that the chemical was of high purity. It was found to be >94% pure and to contain <1% unknown material and <5% hydroxyetylvinyldeuteroporphyrin, the most common contaminant in Hp preparations.

Hp di-ethers were prepared by a modification of the procedures described by Küster and Deihle (1913) and Willstätter and Fischer (1913): Hemin (Sigma Chem. Co.) was dissolved in HBr/acetic acid (specific gravity 1.14, 33%) and allowed to stand for 4 h. After removal of the solvent under reduced pressure, methanol, ethanol, propanol, butanol, amyl alcohol or hexanol was added and allowed to react for 24 h at 50°C. The resulting porphyrins have ether groups at the 2- and 4-positions of the ring and ester groups at the 6- and 7-positions (see Figure 1). The latter groups were removed by treatment with KOH in tetrahydrofuran. The methyl-, ethyl-, propyl-, and butyl-ethers were >90% pure as analyzed by HPLC. Separate runs of each ether were performed, as well as runs of mixtures of all of them to determine their relative retention times. The relation of the retention time to the number of carbon atoms in the substituting alcohol was practically linear (Rimington *et al.*, 1986). In the chromatogram of the amyl-ether two slightly separated peaks appeared (ret. times 30 and 31 min, respectively) related to the fact that commercial amyl alcohol is a mixture of ~87% *iso*-amyl alcohol and 13% amyl alcohol. By the present method it was not possible to prepare the hexyl-ether more than 50% pure. Two major porphyrin contaminants of unknown structure (ret. times 24

**Figure 1** The chemical structure of haematoporphyrin and those of its ethers tested in the present work: $R_1 = H$: haematoporphyrin (Hp); $R_2 = -CH_3$: Hp di-methyl ether; $R_3 = -C_2H_5$: Hp di-ethyl ether; $R_4 = -C_3H_7$: di-propyl ether; $R_5 = -C_4H_9$: Hp di-butyl ether; $R_6 = -C_5H_{11}$: Hp di-amyl ether and $R_7 = -C_6H_{13}$: Hp di-hexyl ether.

Correspondence: J.F. Evensen.
Received 29 September 1986; and in revised form, 19 December 1986.

and 28 min, respectively, as compared with 35 min for the hexyl ether) always appeared. Details of the chemical procedures and of the purity testing are published separately (Rimington *et al.*, 1986).

The porphyrins were brought into sterile, neutral, isotonic, aqueous solution at a concentration of 2.5 mg ml$^{-1}$. First they were dissolved in 0.1 M NaOH, then HCl and NaCl were added to make the solutions neutral and isotonic. HPLC revealed no changes during 2 weeks storage at 4°C. The absorption spectra of all porphyrins used in the present work were recorded, using low concentrations in the mobile HPLC phase. The ratio of the absorbance at 630 nm to that in the Soret band was within 6% similar for all of these porphyrins. Spectral data are given elsewhere (Rimington *et al.*, 1986).

*Animals and tumour system*

Female $C_3D_2F_1$/Bom mice ($C_3H$/Tif ♀ × DBA/2♂, G. Bomholt Gaard, Ry., Denmark) 7–8 weeks old were challenged with a spontaneous C3H/Tif mammary carcinoma by serial transplantation. Tumour for inoculation was obtained by sterile dissection of flank tumours. Macroscopically viable tumour tissue was minced with a pair of scissors and forced through sterile needles of decreasing dimension (19-, 21- and 23-gauge). Finally 0.02 ml of the suspension was injected s.c. on the dorsal side of the foot of the right hind limb by a 25-gauge needle. The mice were assessed regularly and the tumour size measured along 3 orthogonal diameters ($D_1$, $D_2$, $D_3$) every second day, using calipers. The volume was calculated assuming spheroidal geometry $V=(\pi/6)D_1D_2D_3$. When the tumours reached 75–100 mm$^3$ the mice were injected i.p. with 25 mg porphyrin kg$^{-1}$ body weight and irradiated 24 h later. In the cases of HPD and Photofrin II this time between injection and irradiation was found to give an optimal tumour response.

After irradiation each tumour was measured 3 times a week and the results for each porphyrin were pooled to give a mean growth curve. In the regressing phase the size of all tumours on each day was averaged and the mean volume was plotted $\pm 1$ s.e. (vertical bars; Figure 2). Once steady regrowth was established, the analysis was changed: the time taken to reach fixed sizes was determined for each mouse and the mean time was plotted $\pm 1$ s.e. (horizontal bars; Figure 2). The use of the average time to reach a fixed endpoint (horizontal averaging) avoids ambiguities due to different growth rates which may result from variable latency periods as discussed by others (Begg, 1980).

The tumour response was evaluated as the tumour growth time required for a tumour to reach a volume 5 times that at the treatment day. The exponential regrowth phase was evaluated as the tumour doubling time at the point when the tumour was 5 times the treatment volume. These calculations were based on growth curves for each individual mouse (Begg, 1980).

The normal tissue response was evaluated measuring the thickness of a treatment induced oedema in the right hind limb of mice without tumours. These mice were given exactly the same photodynamic treatment as those bearing tumours. After irradiation, the thickness ($T_t$) of the treated and ($T_u$) of the untreated foot was measured 3 times a week for at least 30 days. The normal tissue response was calculated as $(T_t/T_u)-1$. Each treatment group contained 5 mice.

*Irradiation*

Unanaesthetized mice were placed in Lucite jigs with the tumour-bearing leg loosely fixed with tape without impairing the blood flow to the foot. The tumour was then exposed to red light from a Rhodamine 6G dye laser (Spectra Physics 375) pumped by an argon ion laser (Spectra Physics 164). The dye laser was tuned at $630 \pm 5$ nm by use of a monochromator (Jarrel Ash) and the wavelength was regularly checked. The laser beam was defocused by means of a microscope ocular (×12). The fluence rate at the position of the tumour was measured by means of a calibrated thermopile (YSI Kettering model 65A Radiometer) and maintained at 150 mW cm$^{-2}$. The exposure time was 15 min corresponding to an exposure of 135 J cm$^{-2}$.

### Results

The retention time of the Hp di-ethers of an RP18 column increased with increasing length of the carbon chain of the residues $R_{1-7}$ at positions 2 and 4 of the porphyrin (Figure 1; Table I).

In the absence of porphyrins and light the tumours grew exponentially with doubling time of ~2 days. Treatment with porphyrins without light had no effect on tumour growth. Treatment with light alone (15 min, 150 mW) had an insignificant effect on tumour growth and so had treatment with Hp, Hp di-methyl ether and Hp di-ethyl ether combined with light (Figure 2; Table I). Hp di-propyl-, Hp di-butyl- and Hp di-amyl ethers showed increasing efficiencies in this order, while Hp di-hexyl ether was somewhat less efficient than Hp di-amyl ether (Figures 2 and 3; Table I). As expected, Photofrin II was more efficient than HpD, but not significantly more efficient than Hp di-amyl ether (Table I). The same was also true for normal tissue reactions (Figure 4). The reaction with Photofrin II as the sensitizer was quite strong since all the treated mice without tumours lost feet ~8 days after irradiation. Mice treated with Hp di-amyl ether and light showed a similarly strong reaction of their normal tissue at day 1–8 after the irradiation, but all of them gradually healed (Figure 4).

**Table I** Relative efficacy of haematoporphyrin derivatives on tumour growth time and tumour doubling time

| *Porphyrin species* | *Retention time on RP18 column (min)* | *Time to 5× treatment volume (days) mean ± s.e.* | *Doubling time in regrowth phase (days) mean ± s.e.* | *Number of mice* |
|---|---|---|---|---|
| Control (−porphyrin, −light) | | 4.1 ± 0.1 | 2.0 ± 0.1 | 68 |
| Control (−porphyrin, +light) | | 5.7 ± 0.6 | 2.2 ± 0.1 | 16 |
| HP | 6.0 & 6.5[a] | 5.3 ± 0.4 | 2.1 ± 0.1 | 16 |
| HpD | 6–40 | 11.4 ± 0.8 | 2.2 ± 0.2 | 26 |
| PII | 6–40 | 15.9 ± 1.1 | 2.7 ± 0.3 | 15 |
| Hp di-methyl ether | 15 | 4.3 ± 0.2 | 1.9 ± 0.1 | 2 |
| Hp di-ethyl ether | 20 | 4.7 ± 0.4 | 2.0 ± 0.2 | 14 |
| Hp di-propyl ether | 26 | 6.1 ± 0.5 | 2.3 ± 0.1 | 11 |
| Hp di-butyl ether | 29 | 8.4 ± 0.8 | 2.2 ± 0.2 | 7 |
| Hp di-amyl ether | 30 & 31[b] | 14.6 ± 1.0 | 2.3 ± 0.1 | 16 |
| Hp di-hexyl ether | 35 | 12.2 ± 0.7 | 1.9 ± 0.2 | 8 |

[a]Optical isomers. [b]Isomers.

**Figure 2** Growth curves of tumours in mice after treatment with 25 mg sensitizer $kg^{-1}$ body wt and, 24 h later, exposure for 15 min to 150 mW of light at 630 nm. Panel a: ■—■ untreated controls; □—□ haematoporphyrin; ●—● HpD; ○—○ Photofrin II. Panel b: ◇—◇ Hp di-methyl ether; ◆—◆ Hp di-ethyl ether. Panel c: ▼—▼ Hp di-propyl ether; ▽—▽ Hp di-butyl ether. Panel d: ▲—▲ Hp di-amyl ether; △—△ Hp di-hexyl ether. Bars: s.e.

None of the treatments were found to have any significant effect on the tumour doubling time in the regrowth phase (Table I).

### Discussion

The least polar of the Hp-ethers tested in the present work showed a significant efficiency as sensitizers for photodynamic tumour therapy in the present model system. Since the polarity of a compound generally decreases with increasing retention time on a reversed phase HPLC column, the results indicate that for this family of porphyrins the efficiency as sensitizers for PDT increases with decreasing polarity (Figure 3). The reason why our preparation of the Hp di-hexyl ether has a lower sensitizing efficiency than might be expected is probably because it contains significant amounts (50%) of porphyrin impurities with higher polarity than that of the hexyl ether, while the other ether preparations were more than 90% pure. It is also possible that the hexyl-ether which has a low polarity and, therefore, supposedly a low solubility in water, forms large aggregates in aqueous solutions which are of a nature that prevents tumour uptake.

Hp di-amyl ether was equally efficient as Photofrin II in tumour photosensitization (Table I), Photofrin II, Hp di-amyl ether and to a smaller extent HpD, had significant photosensitizing effects also on normal tissue as shown by the induced oedema. Similar oedema may also occur in tumours. Therefore, the effect on tumours for the first few days after irradiation may be underestimated in our measurements. This is also indicated by the fact that in some cases the volume of treated tumours apparently increased more than that of untreated controls during early days after the irradiation (Figure 2a).

The treatment undoubtedly gives rise to damage of the normal tissue in which the tumour grows. This probably affects tumour growth in the early phase after the treatment. However, since the doubling time in the regrowth phase of treated tumours was almost the same as the doubling time of untreated tumours (Figure 2; Table I), we conclude that no large change in the tumour bed occurred.

Although a fluence rate of 150 mW $cm^{-2}$ might be expected to be too low to give hyperthermic effects to the tumour when applied for only 15 min (Gomer *et al.*, 1986) our data indicate that irradiation of unsensitized tumour leads to a slight retardation of the growth (Table I). Thus, it cannot be excluded that hyperthermia contributes slightly to

**Figure 3** The growth delay of treated tumours with different Hp-ethers as sensitizers. Growth delay is given as the difference between the times needed for treated tumours to reach a volume 5 times larger than that at the time of irradiation and the corresponding time for untreated tumours.

**Figure 4** Response of normal tissue (mouse foot) to treatment with HpD ●—●, ○—○ Photofrin II and ▲—▲ Hp di-amyl ether followed by light exposure. Conditions as described for **Figure 2.** The point marked * indicates that at this time the treated feet of all the mice given Photofrin II were lost.

the observed effects. This will be checked further, since it has been shown that moderate hyperthermia may potentiate the effect of PDT (Christensen *et al.*, 1984; Waldow & Dougherty, 1985). However, it is unlikely that slight hyperthermia plays a role that is different for the different sensitizers tested in the present work, and that their relative sensitizing effects would be different under conditions where hyperthermic effects were completely absent. Fluence rates much higher than 150 mW cm$^{-2}$ have been applied in many experimental and clinical studies for the obvious reason to make the exposure time as short as possible.

The most polar sensitizers tested (Hp, Hp di-methyl- and Hp di-ethyl ether) had little or no sensitizing effect (Table I). This is in agreement with the results of Dougherty (1983) who found practically no response of SMT-F mammary tumours in mice to treatment with pure Hp and light.

The present work was supported by The Norwegian Cancer Society (Landsforeningen mot Kreft) and by The Association for International Cancer Research.

### References

BEGG, A.C. (1980). Analysis of growth delay data: Potential pitfalls. *Br. J. Cancer*, **41,** Suppl. IV, 93.

BONNETT, R. & BERENBAUM, M.C. (1983). HPD—a study of its components and their properties. In *Porphyrin Photosensitization,* Kessel, D. & Dougherty, T.J. (eds) p. 241. Plenum Press, New York.

BONNETT, R., RIDGE, R.J. & SCOURIDES, P.A. (1980). Haematoporphyrin derivative. *J.C.S. Chem. Comm.*, **24,** 1198.

CHRISTENSEN, T., WAHL, A. & SMEDSHAMMER, L. (1984). Effects of hematoporphyrin derivative and light in combination with hyperthermia. *Br. J. Cancer*, **50,** 85.

DOUGHERTY, T.J. (1983). Hematoporphyrin as a photosensitizer of tumors. *Photochem. Photobiol.*, **38,** 277.

DOUGHERTY, T.J. (1984). The structure of the active component of hematoporphyrin derivative. In *Porphyrin Localization and Treatment of Tumors,* Doiron, D.R. & Gomer, C.J. (eds) pp 301. Alan R. Liss, New York.

DOUGHERTY, T.J. (1985). Photodynamic therapy. In *Methods in Porphyrin Photosensitization,* Kessel, D. (ed) p 313. Plenum Press, New York.

GOMER, C.J., FERRARIO, A. & MURPHREE, L. (1986). Metastatic potential and natural killer cell activity in mice following porphyrin photodynamic therapy. *Photochem. Photobiol.*, **43,** suppl. 63S.

KESSEL, D., CHANG, C.K. & MUSSELMAN, B. (1985). Chemical, biological and biophysical studies on hematoporphyrin derivative. In *Methods in Porphyrin Photosensitization,* Kessel, D. (ed) p 213.

KÜSTER, W. & DEIHLE, P. (1913). Beiträge zur Kenntnis des Hämatins. *Z. Physiol. Chem.*, **86,** 51.

LIPSON, R.L., BALDES, E. & OLSEN, A. (1961). The use of a derivative of hematoporphyrin in tumor detection. *J. Natl. Cancer Inst.*, **26,** 1.

MOAN, J. (1986). Porphyrin photosensitization and phototherapy. *Photochem. Photobiol.*, **43,** 681.

MOAN, J. & SOMMER, S. (1984). Action spectra for hematoporphyrin derivative and photofrin II with respect to sensitization of human cells *in vitro* to photoinactivation. *Photochem. Photobiol.*, **40,** 631.

RIMINGTON, C., SOMMER, S. & MOAN, J. (1986). Hematoporphyrin ethers I. Generalized synthesis and chemical properties. *Int. J. Biochem.* (in press).

SOMMER, S., MOAN, J., CHRISTENSEN, T. & EVENSEN, J.F. (1984). A chromatographic study of hematoporphyrin derivatives. In *Porphyrins in Tumor Phototherapy,* Andreoni, A. & Cubeddu, R. (eds) p 81. Plenum Press, New York.

WALDOW, S.M. & DOUGHERTY, T.J. (1984). Interaction of hyperthermia and photoradiation therapy. *Radiat. Res.*, **97,** 380.

WILLSTÄTTER, R. & FISCHER, M. (1913). Untersuchungen über Blutfarbstoff. I Abbau des Hämins zu den Porphyrinen. *Zeit. Physiol. Chem.*, **87,** 423.


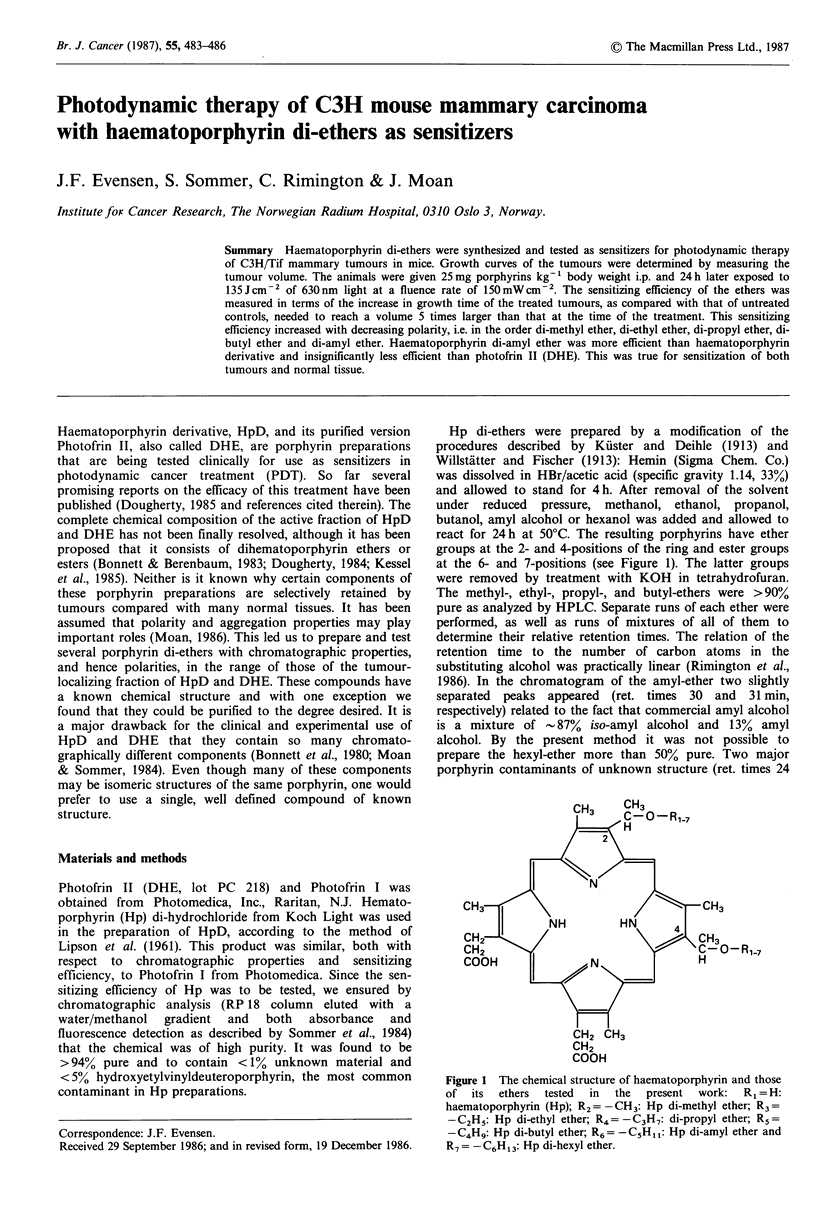

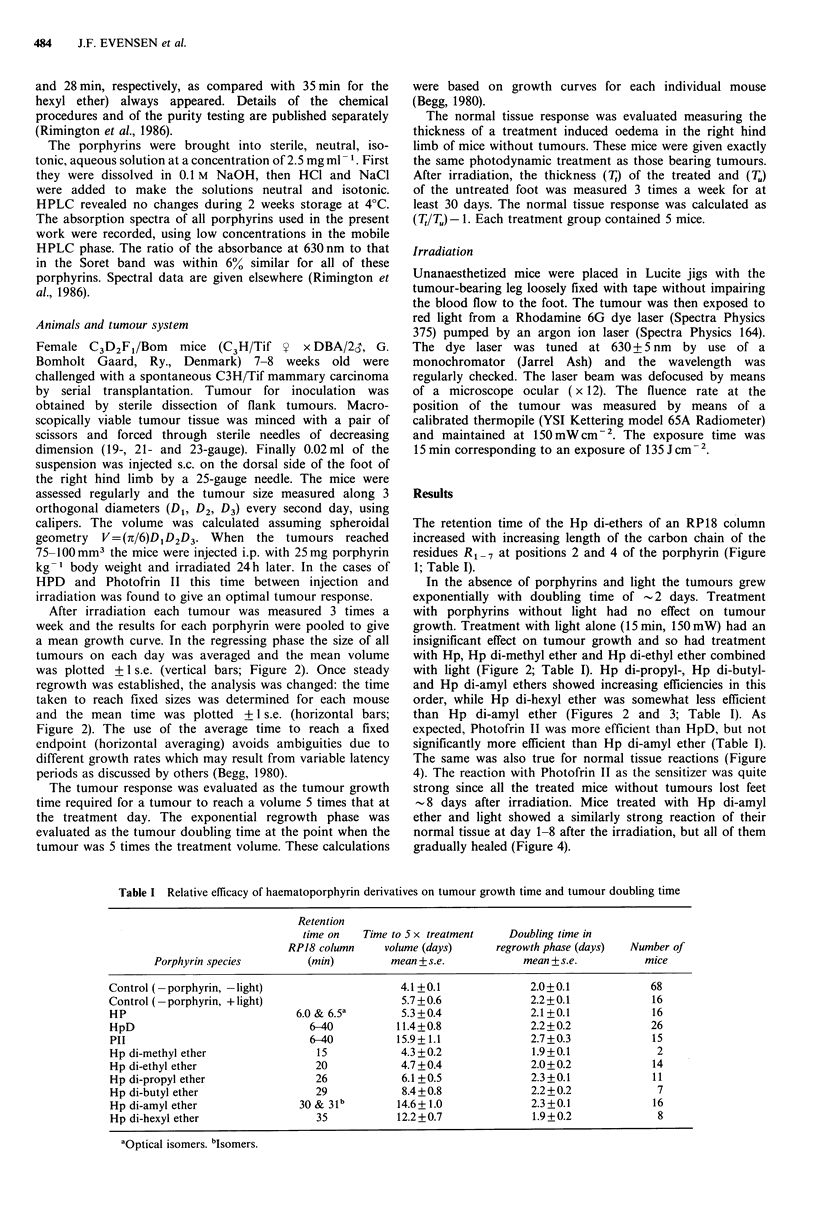

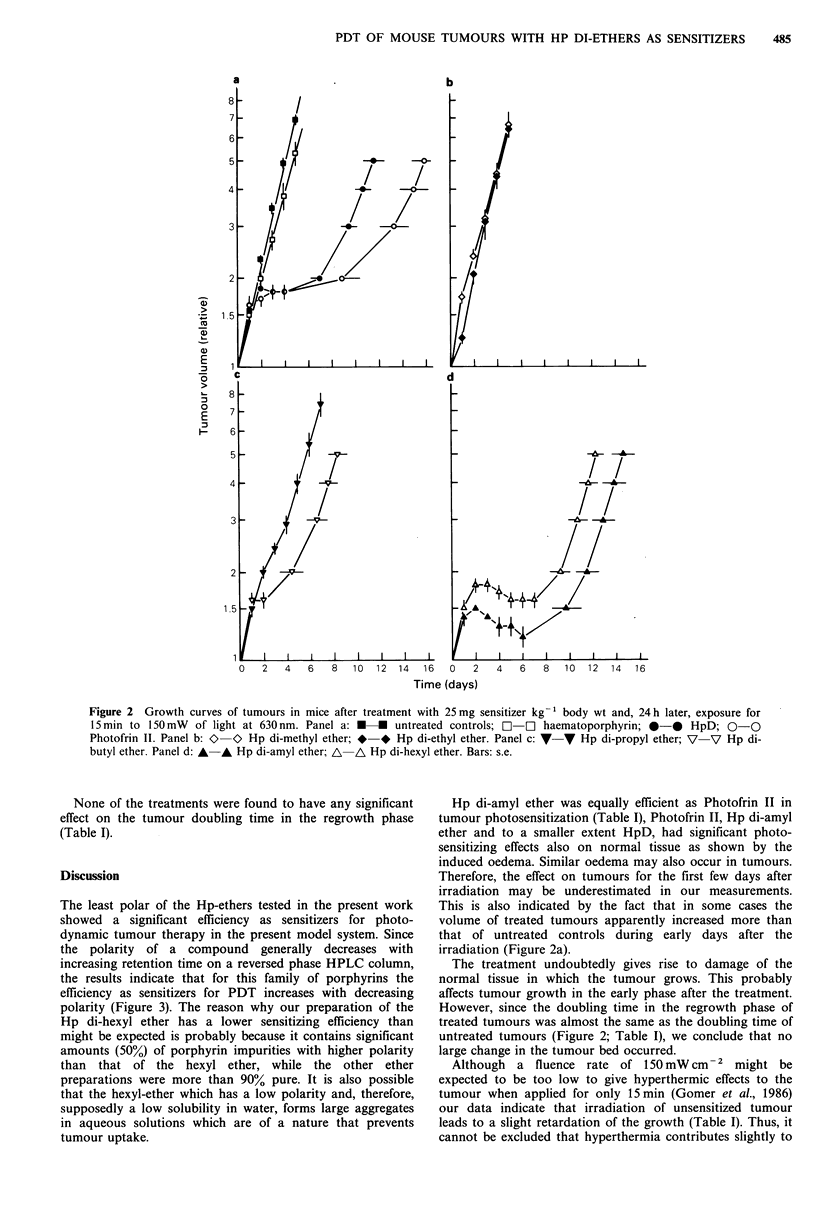

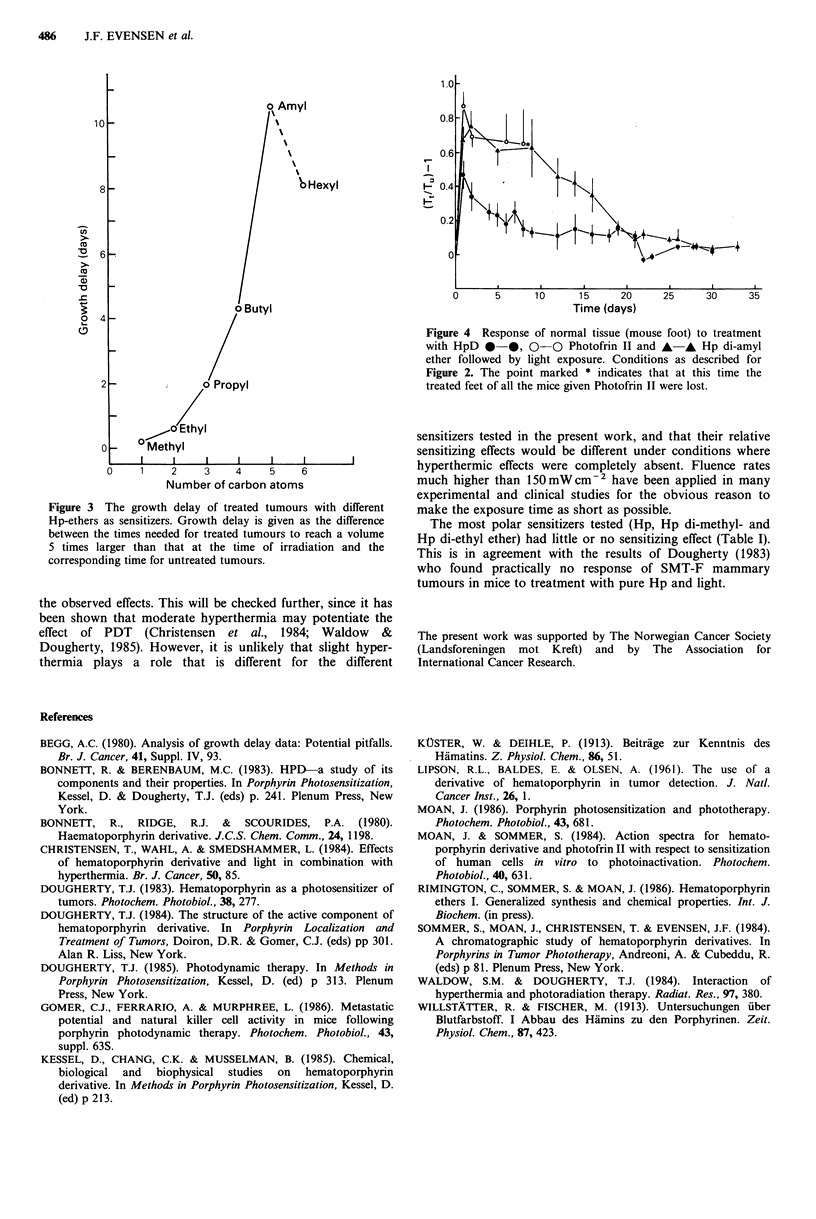


## References

[OCR_00447] Bonnett R., Berenbaum M. C. (1983). HPD - a study of its components and their properties.. Adv Exp Med Biol.

[OCR_00457] Christensen T., Wahl A., Smedshammer L. (1984). Effects of haematoporphyrin derivative and light in combination with hyperthermia on cells in culture.. Br J Cancer.

[OCR_00483] Kessel D., Chang C. K., Musselman B. (1985). Chemical, biologic and biophysical studies on 'hematoporphyrin derivative'.. Adv Exp Med Biol.

[OCR_00493] LIPSON R. L., BALDES E. J., OLSEN A. M. (1961). The use of a derivative of hematoporhyrin in tumor detection.. J Natl Cancer Inst.

[OCR_00498] Moan J. (1986). Porphyrin photosensitization and phototherapy.. Photochem Photobiol.

[OCR_00502] Moan J., Sommer S. (1984). Action spectra for hematoporphyrin derivative and Photofrin II with respect to sensitization of human cells in vitro to photoinactivation.. Photochem Photobiol.

[OCR_00519] Waldow S. M., Dougherty T. J. (1984). Interaction of hyperthermia and photoradiation therapy.. Radiat Res.

